# Design and practice of blended teaching of internal medicine nursing based on O-AMAS effective teaching model

**DOI:** 10.1186/s12909-024-05588-8

**Published:** 2024-05-28

**Authors:** Anyan Duan, Fen Jiang, Ling Li, Qun Li, Wei Chen

**Affiliations:** 1 School of Medicine, Hunan Normal University, Changsha, Hunan China; 2 The Key Laboratory of Model Animals and Stem Cell Biology in Hunan Province, Hunan Normal University School of Medicine, Changsha, China; 3 Nursing Department, The Third Xiangya Hospital, Central South University, Changsha, Hunan China

**Keywords:** O-AMAS teaching model, Blended teaching, Self-directed learning ability, Effective teaching, Internal medicine nursing

## Abstract

**Background:**

Self-directed learning (SDL) ability is the basis for cultivating nursing students’ ability to find and solve problems, lifelong learning, and providing high-quality nursing talents for healthcare. The O-AMAS (Objective, Activation, Multi-learning, Assessment, Summary) model adheres to the teaching philosophy of student-centered, result-oriented, combines the advantages of online and offline teaching, enriching teaching resources and learning channels, diversifying teaching and evaluation methods, and emphasizing integrating and applying knowledge conducive to improving students’ SDL ability and achieving teaching objectives. This study explored the course design, practical, and application effects under the O-AMAS effective teaching model in internal medicine nursing to provide a basis and reference for combining effective teaching models with blended teaching in future nursing courses.

**Methods:**

This study is a self-controlled before-after trial. The participants were 76 nursing undergraduates from Hunan Normal University. This study utilizes the O-AMAS effective teaching model to design internal medicine nursing courses and implement blended online and offline teaching. Main links: The overall course design and application are student-centered, after clarifying macro and micro multi-dimensional learning objectives, with online and offline blended teaching environments activated students’ learning behavior and diversified teachers’ teaching activities, then based on instant and dynamic provide effective feedback; finally, students take the initiate to make a brief and potent summary under the teacher guidance. After the course, a unified assessment of the learning effect of nursing students was conducted, including the evaluation of the SDL ability of nursing students, a final comprehensive evaluation grade, and a teaching satisfaction survey.

**Results:**

The nursing students’ SDL ability scores are higher than before teaching, and the results were statistically significant (*P* < 0.05). The final average comprehensive evaluation grade of nursing students was 78.38 ± 7.12. More than 96% of the students are satisfied with this course.

**Conclusion:**

Applying for internal medicine nursing blended teaching integrated with the O-AMAS effective teaching model is conducive to improving nursing students’ SDL ability, academic grades, and teaching satisfaction.

## Background

The overall purpose of internal medicine course is to cultivate high-quality nursing talents with multi-field adaptive learning ability, cross-field practical ability, and interdisciplinary comprehensive thinking ability to improve the quality of nursing services, meet the increasingly diversified and differentiated nursing needs of people, and achieve the goal of “universal health coverage by 2030” [1–3]. With the rapid update of medical knowledge and technology, nursing students must constantly improve their knowledge, ability, and quality in theoretical teaching and clinical practice, which puts higher requirements for their self-directed learning ability [3–6]. The self-directed learning ability of nursing undergraduates is the ability of undergraduate nursing students to obtain and master the necessary knowledge and skills of nursing services with meta-cognition and objective human and material resources. Its main components are the three abilities of self-management, information, and learning cooperation [7]. Self-directed learning ability is a core competence that equips nursing students with lifelong learning [6, 8–10]. Relevant studies show that the SDL ability enables nursing students to enhance their professional nursing values, self-efficacy, meta-cognitive ability, critical thinking, academic performance, academic resilience, time management tendency, problem-solving ability, health education ability, and resilience [11–17]. However, because nursing students in the process of learning are still influenced by the traditional teaching concept, nursing students’ learning mainly depends on teachers’ classroom teaching, still a passive accept knowledge role, lack of learning initiative, do not understand discipline dynamic and master essential knowledge, especially difficult to use theory knowledge analysis and solve the problem of clinical practice, the nursing students overall SDL ability and SDL readiness is still at a low to medium level [11, 15, 18, 19]. Therefore, educators must reform the traditional teaching mode according to the growth law and the learning needs of contemporary students.

O-AMAS, an online and offline hybrid interactive teaching model, was independently developed and launched by the effective teaching team of Nankai University in 2017. The model has five links: Objective, Activation, Multi-learning, Assessment, and Summary [20]. The model is oriented by learning results and driven by benign interaction. After clarifying multi-dimensional goals, it realizes the teaching objectives and objects are deeply penetrated and participate in the course, advocates student-centered, pays attention to contextualization, gives full play to students’ enthusiasm and initiative, and inspires students to become knowledge builders and problem solvers. The model has been applied in pharmacology, microbial physiology, and community pharmacist training and achieved good results in improving study objects’ self-directed learning ability, academic grades, and satisfaction [21–23].

In recent years, blended learning strategies have become the most potential teaching strategy in nursing education [24]. In 2022, China launched the educational digitization strategy, built an online national intelligent education platform for higher education, and an intelligent overpass for teachers and students to teach and learn [25]. In 2023, China’s Ministry of Education proposed that the digital reform of higher education should pay great attention to content reform and thus provide high-quality educational content to effectively support the steady development of digital education [26]. Therefore, the Internet-based online and offline mixed teaching mode offers new ideas for promoting the construction of nursing “golden courses” [27, 28]. So, this study combines the O-AMAS effective teaching model and online and offline blended teaching to design the internal medicine nursing course. Given the problems existing in the current teaching, analysis and grasp the students learning characteristics and cognitive way, optimize the online teaching resources construction and offline teaching methods, rebuild the internal medical nursing teaching process, use diversified teaching mode, implement effective evaluation and feedback, finally through a brief but potent summary to promote deep learning, fully arouse the students’ learning, improve students’ SDL ability.

## Methods

### Study design

This study is a self-controlled before-after trial.

### Participants

Through cluster sampling, the study defined participants as 76 second year nursing undergraduates from Hunan Normal University, including 11 males and 65 females. All subjects signed an informed consent form before participating in the study.

### Tools and measurements

#### Self-directed learning instrument for nursing students

Self-directed learning instrument for nursing students (SDLI for nursing students) consists of 20 items, using the Likert 5-point scoring method; each item is 1 ~ 5 points, the total score is 20 ~ 100 points, the higher the score, the stronger the self-directed learning ability. The scale contains four dimensions (learning motivation, learning plan and implementation, self-management, and interpersonal communication) [29].

#### Curriculum academic grades

According to the course design, the course team has developed a comprehensive assessment and evaluation system, which combines the process evaluation and final evaluation from online and offline, including classroom performance, chapter test, topic discussion, group activities, clinical internship, and final examination, with each part taking different weights.

#### Teaching satisfaction for nursing student’s questionnaire

The teaching team of this study designed the questionnaire through an extensive review of the literature, examined other satisfaction questionnaires, interviewed nursing students, consulted with academic experts, and then adjusted it according to the course design and practice. The questionnaire comprises eight items on three options (approval, neutrality, and disapproval); the questionnaire assesses aspects such as increased learning interest, engagement, and efficiency. In the current study, the Cronbach’s alpha coefficient was 0.916, indicating a high level of reliability.

### Procedure

Internal medicine nursing is a core and practical clinical course in nursing, but learning it is challenging for most students. On the one hand, the complicated and scattered course content, abstract mechanism, and uneven difficulty hindered learners from activating learning interests and focusing on learning objectives; on the other hand, tight class hours, limited classroom learning resources, and learners cannot reasonably arrange learning resources and formulate learning strategies aren’t conducive to cultivate self-directed learning ability and innovation consciousness [30, 31]. So this study chooses internal medicine nursing to explore the application effects of the O-AMAS effective teaching model and provide a basis and reference for combining effective teaching models with blended teaching in future nursing courses.

**Fig. 1:**
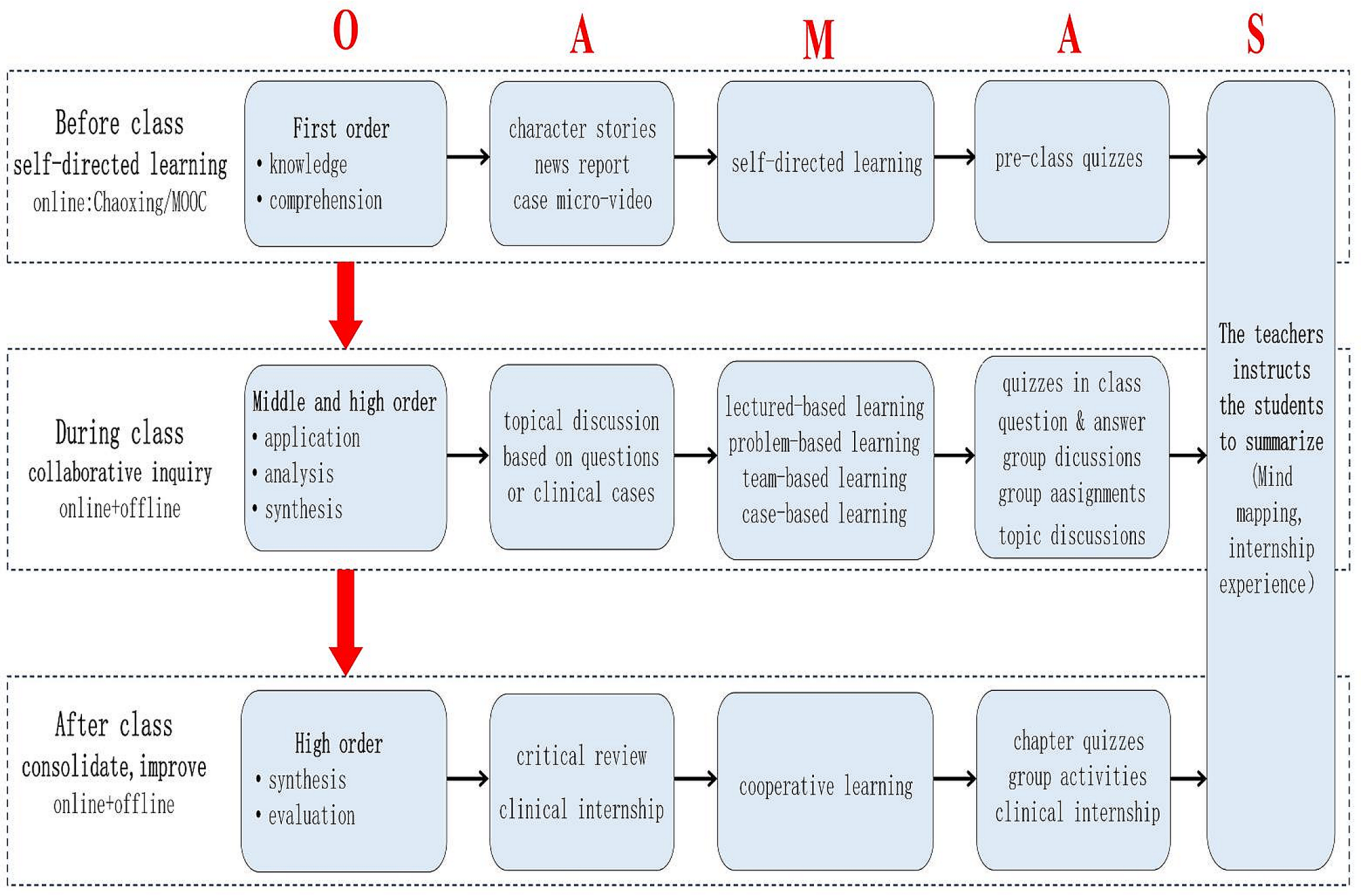
Flow chart of the design of blended teaching of internal medicine nursing based on the O-AMAS effective teaching model

The internal medicine nursing course adopts the O-AMAS effective teaching model to design and mainly includes five parts. The design is summarized in Fig. 1. *Internal Medicine Nursing (6th edition)* is the primary textbook, edited by You Liming and Wu Ying and published by People’s Medical Publishing House. Besides, this course quoted *Internal Medicine (9th edition)* and relevant literature as references, using the MOOC and Chaoxing of online learning applications to assist teaching.

### Assemble a teaching team

This study’s teaching team includes 1 department head, 2 pedagogy experts, 5 internal medicine nursing teachers, and 1 teaching assistant from the Chaoxing learning platform. The department head is responsible for the overall design, implementation, adjustment, and summary of internal medicine nursing; pedagogy experts are responsible for guiding and analyzing students’ learning situation, integrating O-AMAS effective teaching model and blended teaching method design; teachers are responsible for building online teaching platform, course teaching, communicating with students and course evaluation; the teaching assistant is responsible for the smooth combination of online teaching platform and offline courses.

### Combining blended teaching with the O-AMAS model effectively

#### Design effective teaching objectives

The goal of traditional teaching is unclear; most nursing students aim to pass examinations and get a bachelor’s degree certificate, which leads them to neglect often to cultivate their self-directed learning ability. In the long run, it is not conducive for individuals to adapt to the rapidly changing clinical environment and long-term benign development. Therefore, the course team analyzed the students’ learning characteristics and cognitive methods, and under the guidance of the O-AMAS effective teaching model, according to the SMART principles (Specific, Measurable, Achievable, Realistic, Timed) studied and revised the teaching objectives of the internal medicine nursing course, and formulated the macro objectives of the course. The overall teaching objectives of the course: ①Knowledge objectives: Master the basic theory, knowledge, and skills of internal nursing; master the clinical manifestations, nursing diagnosis, nursing plan, nursing measures, and nursing evaluation of common internal diseases; be familiar with the pathologic and physiological change process, diagnostic points and prevention points of common internal diseases; understand the etiology, pathogenesis, and the related frontier research dynamics. ②Ability objectives: Cultivate the ability of nursing students to exert nursing procedures to achieve holistic care; apply interpersonal communication skills to educate medical patients and their families; and learn internal medicine nursing and other related disciplines independently. ③Quality objectives: Cultivate students’ professional spirit and professional quality; cultivate a rigorous and realistic scientific attitude and an innovative scientific spirit; possess a high degree of patriotic feelings and cultural, legal, moral, and professional literacy.

Based on understanding the course’s overall teaching objectives and Bloom’s Taxonomy of educational objectives, this study from six bands, including knowledge, comprehension, application, analysis, synthesis, and evaluation, to design each lesson gradually ascend from low to high orders and student-centered micro-teaching objectives, teachers focus on diversified teaching activities to help students to learn independently and building knowledge system effectively.

Take the Transient Ischemic Attacks(TIA) section as an example: ① Pre-class self-directed learning stage: mainly the first order of knowledge and comprehension, memorizing the brain’s blood supply, and grasping the disease characteristics of TIA. ②Cooperative learning stage in the class: mainly the middle and high order of application, analysis, and synthesis. Students can observe and make initial evaluations, judge the condition of TIA patients, and cooperate with doctors to actively treat, raise existing nursing problems, implement effective holistic care, and provide health guidance to individuals and families. ③Consolidation and promotion stage after class. Based on the high-level evaluation, students can use the knowledge to evaluate whether the nursing measures taken for TIA patients are effective. They can find the existing nursing problems and solutions through books, databases, and other resources.

#### Effective activation to improve students learning interest

Traditional teaching often uses pre-class previews and class tests to activate students, even in some courses that consider the students’ performance an essential part of academic performance. It may have some positive effects, but it also may cause students anxiety and distraction, especially in pure hybrid teaching; students may quickly get answers by intelligent equipment, which not only weakens the students’ interest in learning but can not achieve effective activation. Effective activation requires exciting ways; the activation tools should be closely related to the learning content, and all students participate so that multi-dimensionally activate students’ physical, emotional, and cognition.

This study adopted role-play about clinical reality to activate students’ bodies. Activate students’ emotions by quoting character stories and news reports, such as the diseases that exist in real heroes and ordinary families taking their precious lives, leading to the country and the family loss of talents and relatives to activate students’ empathy; quote positive clinical cases to activate students responsibility, such as healthcare workers save countless patients struggling in disease through solid fundamental knowledge and meticulous clinical work, the teacher praised students’ and created a positive learning atmosphere to activate students’ confidence based on the student’s performance. Presented clinical micro-video and conducted thematic discussions to activate students’ cognition. For example, the leukemia teaching combined with the teaching objectives and content of this chapter, when talking about “acute leukemia,” the teacher through micro-video to initiate a discussion topic, “Does the blood of patients with leukemia turn white?” to motivate student learning interest and enthusiasm, so that students can quickly get into the learning state. The teacher further used brainstorming to guide the students to state whether the blood of leukemia patients will turn white and analyze the causes on the Chaoxing learning platform; the classroom screen will display each student’s ideas to fully mobilize the students to participate actively in the classroom with enthusiasm.

#### Diversified teaching methods to enhance students’ motivation

Due to the differences between nursing students in personality, learning habits, learning foundation, and learning methods, teachers are required to build new and diversified learning methods to meet the learning needs of most students. This study, based on students’ learning conditions and specific courses, flexibly adopts multiple teaching methods, such as lectured-based learning(LBL), team-based learning(TBL), problem-based learning(PBL), and case-based learning(CBL) etc. A variety of teaching methods are interspersed. At the same time, teachers guided students to experience various learning methods, such as receptive learning, cooperated and explored learning, independent learning, etc. Suppose students must pass the pre-class quizzes to reach the low-order knowledge and comprehension teaching objectives. In that case, the teachers will focus on the class’s LBL and PBL teaching methods, or the teachers will take students as the leading role, application and analysis as the primary teaching objectives, and CBL and TBL as the central teaching methods.

Take acute coronary syndrome and cerebrovascular disease as an example; since students have comprehended the definition and characteristics of the disease, so the teacher took the progressive cases as the main line to present different scenarios of progressive cases gradually. As the disease progresses, it gradually leads to core knowledge points that include the cause of the disease, clinical manifestations, treatment, and nursing measures, etc., and further through teaching strategies such as questioning, enlightening thinking, and group discussion, encourage and guide students to independently explore the implicit knowledge behind the case scenario (such as the causes and inducements of the disease, typical clinical manifestations, and laboratory changes that contribute to identifying the disease, judgment disease of changes, observe treatment efficacy and care adverse reactions), so then guide students to actively participate in the class, more profoundly and systematically understand the internal logical relationship of disease occurrence and development. Finally, according to the feedback from testing, teachers organized students to discuss the frontier or hot topic of clinical nursing. They guided students to track the latest research results and hotspots of nursing. Besides, in the after-class clinical internship, cooperative learning is the primary method; students enter the ward in groups to collect data, and the teachers observe by the bed and give timely guidance and supplements. After the nursing assessment, the students broached the nursing problems and nursing measures according to the case under the teachers’ guidance, evaluated the implementation effect of the nursing measures, and broached the improvement plan. At the end of the internship, the students completed the internship report, which included nursing medical records and experience.

#### Effective assessment improve teaching effect

The three elements of effective assessment are assessment design, implementation, and feedback. An effective assessment is not a simple score but an effective feedback activity corresponding to the teaching objectives to promote students’ learning effect. This study combines process assessment and final assessment, attaches importance to process assessment and practical ability assessment, continues to assess teaching activities according to teaching objectives, timely adjusts and improves teaching strategies based on students’ feedback, and the assessment runs through the whole online and offline teaching activities. The assessment methods include before and after classroom evaluation, classroom questions and answers, online engagement in the topic discussion, chapter tests, group activities, and clinical internship performance, etc.; the assessment content includes whether the low-order goals (knowledge and comprehension) and the high-order goals (application, analysis, synthesis, and evaluation) can be achieved. The assessment system of internal medicine nursing is presented in Table 1. For example, this study adopted multiple assessment forms to achieve practical evaluation and feedback, such as group discussion based on clinical progressive cases, topic discussion, and thinking questions. Students can realize how much knowledge they can master in classroom learning and what aspects they need to make up for the deficiencies; it effectively facilitates students to adjust learning objectives timely. Meanwhile, teachers can also analyze the effect of classroom teaching from a multi-dimensional perspective to improve and further promote effective teaching.

**Table 1:** Assessment system of internal medicine nursing course

Assessment design	Process assessment					Final assessment
Methods	question-answer process in class	chapter quiz	topic discussion	group activities	clinical noviceship	final exam
Ratio	5%	5%	10%	10%	10%	60%
Forms	offline	online	online + offline	online + offline	offline	offline

#### Brief summary to promote in-depth learning

This stage is often at the end of a class. After students focus on learning the vital and challenging points of knowledge in class, they tend to become lax at this stage. Therefore, a short and powerful summary is needed to help students connect the key learning content of a class. With students as the main body, teachers guide students in summarizing the classroom content and integrating the learning emphases. Based on the Chaoxing learning platform, it automatically generates summative hot words, or students summarize the teaching content to form mind mapping to help students review what they have learned, further consolidate, reflect, and deepen knowledge.

### Statistical data analysis

Entered and analyzed data using the SPSS 23.0 statistical software, measurement information was expressed as mean ± standard deviation ($\overline {\rm{X}} {\rm{  \pm  S}}$), and a paired-sample t-test was used to compare students’ self-directed learning ability before and after teaching. The test level is α = 0.05, 1-β = 0.9, *p*-value<0.05 was considered statistically significant.

### Ethical consideration

Informed consent was obtained from the study participants before they were enrolled in the study. Ethical approval was obtained from the Institutional Review Committee of Hunan Normal University School of Medicine before commencing the study. (Ref no 2,023,415, dated 10th February 2023)

## Results

### Results of final comprehensive grades

In the teaching process, the Chaoxing learning platform, the classroom learning engagement, and the after-class assessment together form the process grades, which combine the final exam score to get the final comprehensive grades. The course passing rate is higher, as recorded in Table 2.

**Table 2:** Results of the scores of different sources of grades ($\overline {\rm{X}} {\rm{  \pm  S}}$, points)

Source of grades	Scores
Process grades	91.69 ± 8.03
Final exam score	69.51 ± 9.74
Final comprehensive grades	78.38 ± 7.12

### Results of evaluation of teaching satisfaction

After teaching, using a self-made questionnaire to evaluate the teaching effect, 100% of students gave feedback; more than 90% of students think it is helpful to promote pre-class preview and after-class review and consolidate; more than 80% of students think it helps to stimulate learning interest and enthusiasm, mobilize learning initiative, help adjust learning methods and improve learning efficiency, and teachers can timely feedback; the course satisfaction rate for all students reached 96.1%. The satisfaction results are presented in Table 3.

**Table 3:** Results of students’ evaluation of teaching satisfaction(%)

Evaluation content	Agree	Neutral	Disagree
1.Clarify learning goals	73(96.1)	1(1.3)	2(2.6)
2.Stimulate the interest and enthusiasm of learning	61(80.2)	11(14.5)	4(5.3)
3.Mobilize the learning initiative	64(84.2)	8(10.5)	4(5.3)
4.Adjust learning methods and improve learning efficiency	64(84.2)	9(11.8)	3(3.9)
5.Review and consolidate the knowledge learned	72(94.8)	2(2.6)	2(2.6)
6.The learning activities arranged by the teacher can make more involved in study	64(84.2)	9(11.8)	3(3.9)
7.Teachers can answer questions in time, or evaluate and feedback learning behavior in time	67(88.2)	8(10.5)	1(1.3)
8.Very generally satisfied with the course	73(96.1)	1(1.3)	2(2.6)

### Results of comparison of SDL ability before and after teaching

The after-teaching was superior to the before-teaching in the total score of nursing students’ SDL ability (*P* < 0.05). The scores of nursing students’ SDL ability were improved in four dimensions, especially in learning motivation, planning and implementation, and self-management (*P* < 0.05). The SDL ability scores before and after teaching are presented in Table 4.

**Table 4:** Results of comparison of SDL ability scores between before-teaching and after-teaching ($\overline {\rm{X}} {\rm{  \pm  S}}$, points)

Time	Learning motivation	Planning and implementation	Self-management	Interpersonal communication	Total points
Before-teaching	20.88 ± 2.97	18.86 ± 3.12	12.99 ± 1.85	13.53 ± 2.08	66.25 ± 6.37
After-teaching	22.17 ± 3.40	20.75 ± 3.86	13.79 ± 2.37	14.22 ± 2.54	70.93 ± 10.29
*t*	-2.492	-3.328	-2.324	-1.854	-3.373
*P*	0.014	0.001	0.021	0.066	0.001

## Discussion

Existing research results show that the students’ SDL ability is positively associated with health education ability, clinical practice behavior, and learning motivation [17, 32–34] and negatively associated with study burnout and academic stress [35], so improving the nursing students’ SDL ability is beneficial to promote the nursing students’ better master theoretical knowledge and operation skills, integrate into the clinical work, promote professional identity, cultivate consciousness and ability of lifelong learning, improve clinical nursing quality in all aspects, meet the growing social health needs [36]. The O-AMAS effective teaching model emphasizes the educational philosophy of “student-centered and result-oriented development” in all teaching activities [20]. This study is based on the digital educational trend, combined with the national Massive Open Online Courses(MOOC), the school Small Private Online Course (SPOC) teaching resources, the Chaoxing learning platform, and the Wisdom Tree Platform teaching tools, which scientifically cover pre-class preview, class teaching, and post-class improve three teaching link, designed and practiced blended teaching of internal medicine nursing teaching based on O-AMAS effective teaching model. Establishing a multi-level oriented multi-dimensional goal in line with Bloom’s Taxonomy of educational objectives; Quickly activating the learning interest and behavior from the three aspects of students’ physical, emotional, and cognitive; adopting multiple teaching methods to guide nursing students learning autonomously based on the needs of diagnostic theory and experimental manipulation; combined with the Chaoxing online learning platform for effective measurement and assessment, multi-dimensional analysis, process evaluation and dynamic feedback on the teaching and learning behaviors generated during the course teaching process, to promote the timely improvement of teaching and learning; finally, teachers take the students as the main body and guide students to generate summarizing hot words or mind mappings, help students review what they have learned, explore the relevant scientific research frontiers, and further consolidate and deepen their reflections, improve the coherence and efficiency of nursing students both in and out of class, reduce the learning burden of nursing students, so as to promote the improvement of learning enthusiasm and independent learning ability.

Blended teaching based on the O-AMAS effective teaching model is beneficial to improve the academic performance of nursing students. After teaching, the student’s average academic score was 78.38 ± 7.12 points, which was higher than the academic score of the previous students. More than 96% of students are generally satisfied with this course, and more than 80% believe it can stimulate their interest in learning, mobilize their learning initiative, and increase their learning investment. Moreover, the SDL ability of nursing students was higher than before class, with statistically significant differences (*p* < 0.001). These results show that the O-AMAS effective teaching model helps to analyze and grasp students’ learning characteristics and cognitive mode, clarify multi-dimensional learning objectives, effectively and quickly activate the students’ learning behaviors and interests, and significantly improve students’ participation in class. The application of the online curriculum platform for teachers and students to communicate has provided more opportunities and diverse methods; while improving feedback efficiency, it can effectively promote the adjustment and improvement of teaching and learning methods. In this process, teachers’ pedagogy improved, and students effectively realized the enhancement of knowledge level and clinical ability. Both sides progress to complete the overall goal of the course and develop a harmonious relationship. Therefore, students achieved excellent academic performance and were given a higher teaching evaluation. In the post-epidemic era, Shen Bingzheng et al. based on the O-AMAS teaching model and flipped classroom, developed an online continuing training program, effectively improved community pharmacists’ SDL ability, professional competence online, and received a high evaluation of teaching satisfaction [23]; Luo PeiPei et al. adopted the results-oriented effective teaching mode (O-AMAS) to guide the clinical nursing teaching of undergraduates in cardiovascular internal medicine and improved the nursing students of theoretical scores, comprehensive skills test score, and evaluation of clinical teaching effect [37]. Wang Xiaojun et al. applied the O-AMAS effective teaching model in the health assessment course, which effectively improved the teaching effect, improved the teaching evaluation of teachers and students, and cultivated students’ independent learning ability [38]. Therefore, the O-AMAS effective teaching model is helpful in improving students’ self-learning ability, academic performance, and teaching satisfaction and is also suitable for nursing teaching in different settings.

This study further found that the self-directed learning instrument for nursing students of the three dimensions’ total scores (learning motivation, planning and implementation, self-management) were higher than before teaching, with statistically significant differences (*p* < 0.05), which can be attributed to that the teaching model expand the cognition of learning from the starting point of teaching, let students get rid of the idea of learning for the sake of examination, and realize that the course of internal medicine nursing can help them master knowledge, improve their ability, and establish good professional ethics, to correct, activate and maintain the learning motivation of learners [39]; adhere to the student-centered from beginning to end, online and offline, and provides external learning conditions and resources that suitable for students’ learning paths, starting from the activation of existence, then using self-improvement as the intermediary, students to formulate and implement learning plans according to their own situation in the support of rich teaching resources and various teaching methods, finally promote individual self planning and implementation ability [40]. Besides, combining the online learning platforms of MOOC and the Chaoxing Learning platform to multi-dimensionally analyze, dynamically evaluate, and provide feedback improves students’ ability to actively think, explore, and build knowledge systems. Supervise and manage students’ online learning situations and include usual performances as process assessment data, which improves students’ self-management ability to a certain extent [41–44]. Compared with traditional face-to-face teaching and simple online teaching, the blended teaching mode that integrate online resources and clinical case has more positive effects on student’s academic performance [45, 46], self-directed learning ability [47, 48], learning interest [49], motivation [50], and satisfaction [51, 52]. It is also worth noting that the interpersonal communication skills of nursing students improved compared with before teaching, but the result did not have statistical significance. In terms of interpersonal communication, when applying the O-AMAS effective teaching model in this course, the teaching process takes students as the main line and teachers as the guidance, but online learning mainly focuses on students’ independent learning, with fewer chapters on peer learning, team-based learning, and learning feedback between peers. Therefore, there is no noticeable improvement in students’ SDL ability in interpersonal communication. In the future, education must focus on cultivating students’ confidence to improve interpersonal communication.

## Conclusion

The results of this study show that the design of blended teaching of internal medicine nursing based on the O-AMAS effective teaching model has an explicit level, rich content, a wide range of applications, more than 100 effective interactive methods, and a variety of teaching methods, teaching techniques, teaching organization and management complements each other, which can effectively improve students’ academic performance and SDL ability, further stimulating students’ enthusiasm for learning to encourage students to learn more actively and effectively. Students’ high satisfaction with the course also promotes the establishment of harmonious relationships between teachers and students and the realization of course objectives; the course objectives and teaching objectives are successfully realized and deeply penetrate the teaching process, the teaching objects are deeply involved, the teaching methods have rules to follow, and the teaching effect is visible and controllable. This study has limitations in the number of courses applied, class hours, and sample size, which need to be improved in future studies. Therefore, nursing teaching needs to innovate the classroom teaching mode and optimize the teaching process constantly to promote the development of students’ self-directed learning ability, improve students’ innovation capacity, and lay a talent foundation for the sustainable development of China’s health cause.

## Data Availability

The datasets used and/or analyzed during the current study are available from the corresponding author on reasonable request. The data are not available publicly due to privacy.

## Abbreviations

SDL: Self-directed learning
SDLI for nursing students: Self-directed learning instrument for nursing students
TIA: Transient Ischemic Attacks
LBL: Lectured-based learning
TBL: Team-based learning
PBL: Problem-based learning
CBL: Case-based learning
MOOC: Massive Open Online Courses
SPOC: Small Private Online Course
